# Molecular Characterization and Target Prediction of Candidate miRNAs Related to Abiotic Stress Responses and/or Storage Root Development in Sweet Potato

**DOI:** 10.3390/genes13010110

**Published:** 2022-01-06

**Authors:** Li Sun, Yiyu Yang, Hong Pan, Jiahao Zhu, Mingku Zhu, Tao Xu, Zongyun Li, Tingting Dong

**Affiliations:** 1 Institute of Integrative Plant Biology, School of Life Science, Jiangsu Normal University, Xuzhou 221008, China; 15165542391@163.com (L.S.); hongzhuang00140020@163.com (Y.Y.); f2694635698@gmail.com (J.Z.); mingkuzhu007@126.com (M.Z.); 2 Jiangsu Key Laboratory of Phylogenomics & Comparative Genomics, School of Life Sciences, Jiangsu Normal University, Xuzhou 221008, China; A2627147086@126.com (H.P.); xutao_yr@126.com (T.X.)

**Keywords:** miRNA, abiotic stress, hormone, storage root, sweet potato

## Abstract

Sweet potato is a tuberous root crop with strong environmental stress resistance. It is beneficial to study its storage root formation and stress responses to identify sweet potato stress- and storage-root-thickening-related regulators. Here, six conserved miRNAs (miR156g, miR157d, miR158a-3p, miR161.1, miR167d and miR397a) and six novel miRNAs (novel 104, novel 120, novel 140, novel 214, novel 359 and novel 522) were isolated and characterized in sweet potato. Tissue-specific expression patterns suggested that miR156g, miR157d, miR158a-3p, miR167d, novel 359 and novel 522 exhibited high expression in fibrous roots or storage roots and were all upregulated in response to storage-root-related hormones (indole acetic acid, IAA; zeaxanthin, ZT; abscisic acid, ABA; and gibberellin, GAs). The expression of miR156g, miR158a-3p, miR167d, novel 120 and novel 214 was induced or reduced dramatically by salt, dehydration and cold or heat stresses. Moreover, these miRNAs were all upregulated by ABA, a crucial hormone modulator in regulating abiotic stresses. Additionally, the potential targets of the twelve miRNAs were predicted and analyzed. Above all, these results indicated that these miRNAs might play roles in storage root development and/or stress responses in sweet potato as well as provided valuable information for the further investigation of the roles of miRNA in storage root development and stress responses.

## 1. Introduction

MicroRNAs (miRNAs) are a series of single-stranded sRNAs that usually consist of 20–24 nucleotides [1,2]. They are produced by complex biological processes. First, pri-miRNAs are transcribed by miRNA-coding genes. Then, pri-miRNAs are cut by the drosha–DGCR8 complex to produce pre-miRNAs that have hairpin secondary structures. Subsequently, pre-miRNAs are identified and cut by RNase Dicer, and mature miRNA as well as miRNA* are produced. Finally, most miRNA*s are degraded, and the miRNAs form RISC [3,4].

The main function of miRNAs is to play crucial roles in post-transcriptional regulation [5]. In plants, miRNAs are related to plant development and resistance. For instance, miR319 regulates the formation of leaf serrations by inhibiting *TCP* (*TEOSINTEBRANCHED1*, *CYCLOIDEA* and *PROLIFERATING CELL NUCLEAR ANTIGEN BINDING FACTOR*) genes [6]. miR172 functions in the determination of flower development and flowering by targeting *AP2* (*APETALA2*) and/or *AP2-like* genes [7]. miR390 regulates lateral root formation and growth by negatively regulating auxin response factors (ARFs) [8]. Additionally, a set of miRNAs have been confirmed to play essential roles in plant stress resistance. miR164 is characterized as an important player in salt and drought stress resistance by regulating the expression of NAC genes [9,10]. miR398 could mediate the accumulation of ROS and the response to high-temperature stress by impacting its target genes: *CSDs*, *CCS1* and/or *COX5b-1* [11,12]. Moreover, plants that overexpressed miR319 exhibited improved tolerance to salt stress [13].

Sweet potato (*Ipomoea batatas* (L.) Lam.) is a tuberous root crop with strong environmental stress resistance and poor soil tolerance. It is beneficial for finding regulators related both to storage root development and stress resistance to investigate the characteristics of sweet potato; research has confirmed that miRNAs play crucial roles in both aspects [14,15]. Thus, we predict that there may be some miRNAs related to both sweet potato storage root development and stress resistance. In recent years, several miRNAs have been identified by high-throughput sequencing in sweet potato. Saminathan and Reddy reported that 32 known miRNAs and 25 novel miRNAs were differentially expressed during drought and that CO_2_ stresses were identified in sweet potato by small RNA sequencing [16]. A total of 190 conserved miRNAs and 191 novel miRNAs were identified from sweet potato storage roots under cold stress treatment [17]. Fifty-one miRNAs were markedly induced and 76 miRNAs were significantly reduced in sweet potato leaves; 13 miRNAs were strikingly upregulated and nine miRNAs were obviously downregulated in sweet potato roots by salt stress treatment [18]. The sRNA sequencing results of He et al. reported 121 differentially expressed miRNAs between white flesh sweet potato (Xushu 18) and purple flesh sweet potato (Xuzishu 3) [19]. Above all, there have been many high-throughput sequencing data on sweet potato miRNA. Unfortunately, only a few of them have been analyzed deeply. The relationship between miRNAs and sweet potato resistance and/or storage root thickening is largely unknown.

In this study, six conserved miRNAs and six novel miRNAs were isolated and characterized in sweet potato. The structures of the novel miRNAs and the expression profiles of all twelve miRNAs were investigated. In addition, the expression patterns of these twelve miRNAs under abiotic stresses (salt, dehydration and cold as well as heat stress) and development/stress-related hormone treatments (IAA, indole acetic acid; ZT; zeaxanthin; GAs, gibberellin; and ABA, abscisic acid) were analyzed. Moreover, the candidate targets of these miRNAs were predicted. These results provide valuable information for the further investigation of miRNA roles in storage root development and stress responses of sweet potato.

## 2. Materials and Methods

### 2.1. Plant Materials

Sweet potatoes (Xushu 22) were grown in a greenhouse at 18–28 °C under a long-day photoperiod (16/8 h, light/dark). For organ-specific expression profiling of miRNAs and target genes, the tissues of stems, leaves, fibrous roots and storage roots in different stages were collected and promptly frozen in liquid nitrogen and stored at −80 °C until required.

### 2.2. Identification of Sweet Potato miRNAs

Six known miRNAs (miR156g, miR157d, miR158a-3p, miR161.1, miR167d and miR397a) and six novel miRNAs (novel 104, novel 120, novel 140, novel 214, novel 359 and novel 522) were identified by using our high-throughput sequencing analysis of sweet potato [20]. Sweet potato fibrous roots and four different developmental stages of storage roots were collected for small RNA sequencing. Twelve miRNAs that differentially expressed in fibrous roots compared with storage roots were chosen.

### 2.3. Predicted Hairpin Secondary Structures of Novel miRNAs

The hairpin secondary structures of novel miRNAs were predicted by RNAfold (http://rna.tbi.univie.ac.at//cgi-bin/RNAWebSuite/RNAfold.cgi, accessed on 17 December 2021). For fold algorithms and basic options, “minimum free energy (MFE) and partition function” and “avoid isolated base pairs” were used.

### 2.4. Abiotic Stress and Hormone Treatments

Abiotic stress (salt, dehydration and cold as well as heat treatments) and plant hormone treatments (IAA, ZT, ABA and GAs) were performed as described by Dong et al. [21]. Sweet potato plants with 5–6 leaves and 8–10 cm adventitious roots were chosen and cultured in water. For the salt, dehydration and hormone treatments, 150 mM NaCl, 100 mM PEG (polyethylene glycol), 100 μM IAA, 100 μM ZT, 100 μM ABA and 100 μM GAs were used and the plants were cultured in a greenhouse. For the cold and heat treatments, the pants were cultured in water at 4 °C and 40 °C. Then, adventitious roots were collected after 0, 1, 12, 24 and 48 h for analysis.

### 2.5. QRT-PCR Analysis of miRNAs

Total RNA was isolated from sweet potato leaves (leaves obtained from three-month-old plants), stems (stems obtained from three-month-old plants) and roots at five stages (FR, fibrous roots; D1, 1 cm storage roots; D3, 3 cm storage roots; D5, 5 cm storage roots; and D10, 10 cm storage roots) by a RNAprep Pure Plant Plus Kit (Polysaccharides & Polyphenolics-rich; TIANGEN, Beijing, China). Reverse transcription and qRT-PCR were performed as described by Tang et al. [20]. An adaptor (5′ GTC GTA TCC AGT GCA GGG TCC GAG GTA TTC GCA CTG GAT ACG AC 3′) was added to the 3′ end of the miRNAs through a reverse transcription program; the reverse transcription of the miRNAs was performed by a PrimeScript™ RT reagent kit with gDNA Eraser (TaKaRa, Dalian, China). qRT-PCR was performed by using TB Green™ Premix Ex Taq™ II (TaKaRa, Dalian, China) and CFX96™ Real-Time System (Bio-Rad, Hercules, CA, USA) with the following procedures: 95 °C for 30 s, then 95 °C for 5 s and 60 °C for 40 s for 40 cycles. The primers used for miRNA qRT-PCR analysis are listed in Table S2. Sweet potato *ARF* (ADP-ribosylation factor) was used as the reference gene for the normalization of gene expression [22].

### 2.6. Target Prediction and Analysis

The target genes of the miRNAs were predicted by combined analysis of RNA-seq and sRNA-seq, and a set of potentail targets of each miRNA have been identified [20]. Then, one predicted target of each miRNA was chosen and its expression in sweet potato leaves, stems and roots was analyzed by qRT-PCR. The methods used for the qRT-PCR of the target genes were performed by using TB Green™ Premix Ex Taq™ II (TaKaRa, Dalian, China) and a CFX96™ Real-Time System (Bio-Rad, Hercules, CA, USA) with the procedures below: 95 °C 30 s, then 95 °C 5 s, 60 °C 40 s for 40 cycles. Sweet potato *ARF* was used as the reference gene and the primers used for target qRT-PCR analysis are listed in Supplementary Materials Table S2.

### 2.7. Statistical Analysis

The data were statistically analyzed by SPSS software (IBM Corp., Armonk, NY, USA) with an ANOVA (one-way analysis of variance), and differences in means were determined to be significant by a Dunnett’s test at *p* < 0.05 and *p* < 0.01. Additionally, to make the statistical inference more convenient and reasonable, a log10 transformation of the expression data was performed.

## 3. Results

### 3.1. Isolation and Structure Analysis of Sweet Potato miRNAs

**Figure 1:**
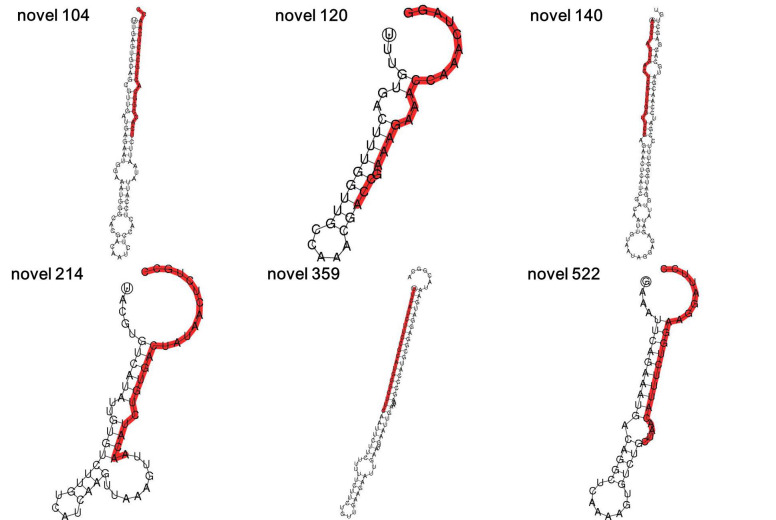
Hairpin secondary structures of novel miRNAs. The hairpin secondary structures of novel 104, novel 120, novel 140, novel 214, novel 359 and novel 522. The mature sequences of the six novel miRNAs are marked red.

Six known miRNAs (miR156g, miR157d, miR158a-3p, miR161.1, miR167d and miR397a) and six novel miRNAs (novel 104, novel 120, novel 140, novel 214, novel 359 and novel 522) were identified and selected by high-throughput sequencing analysis of sweet potato. The mature sequences and pre-miRNA sequences of these miRNAs are listed in Table S1. Then, the secondary structures of the novel pre-miRNAs were predicted. The results showed that all six novel miRNAs could form hairpin structures (Figure 1).

### 3.2. Organ-Specific Expression of miRNAs in Sweet Potato

**Figure 2:**
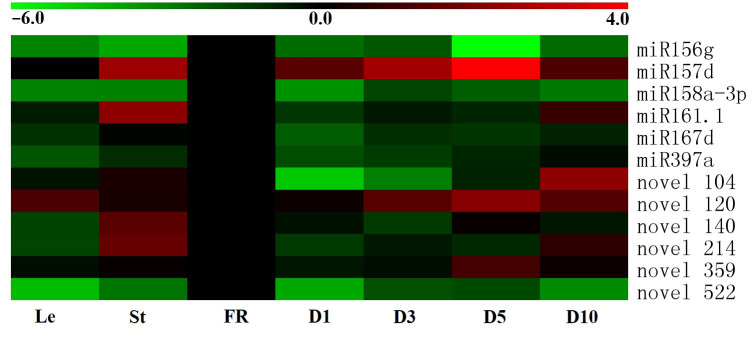
Expression profiles of six conserved and six novel miRNAs in different tissues of sweet potato. The tissues were obtained from Xushu 22 and the expression profiles of the miRNAs were detected by qRT-PCR. Log2-transformed fold-change data were used to create a heatmap via MeV4.9. The relative expression of FR was normalized to 0. Le, leaves obtained from three-month-old plants; St, stems obtained from three-month-old plants; FR, fibrous roots; D1, 1 cm storage roots; D3, 3 cm storage roots; D5, 5 cm storage roots; and D10, 10 cm storage roots.

Organ-specific expression profiles of the miRNAs were analyzed by qRT-PCR. The results showed that the twelve miRNAs exhibited different levels of expression in different sweet potato tissues (Figure 2). miR156g, miR158a-3p and novel 522 exhibited high expression in fibrous roots of sweet potato, while lower levels of expression were observed in other tissues (Figure 2). Similar to these three miRNAs, miR167d and miR397a not only exhibited high expression in fibrous roots but were also expressed highly in stems and mature storage roots, respectively (Figure 2). In contrast, miR157d, novel 120 and novel 359 were expressed highly in the D5 stage of storage roots than in other tissues, and novel 104 showed high expression in D10 stage of storage roots (Figure 2). In addition, the predominant expression of miR161.1, novel 140 and novel 214 was detected in stems, while a small number of these miRNAs were accumulated in other tissues (Figure 2). These sundry tissue-specific expression profiles suggested that the twelve miRNAs might play multiple functions during the growth and development of sweet potato.

### 3.3. The miRNAs Participate in Resistance to Various Abiotic Stresses in Sweet Potato

**Figure 3:**
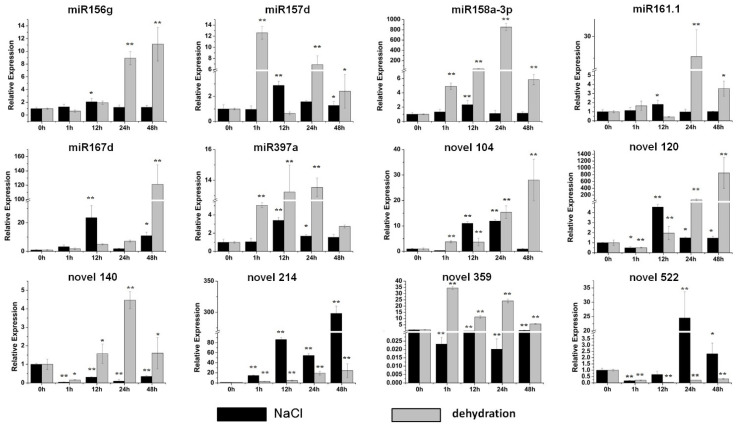
Expression profiles of six conserved and six novel miRNAs under salt and dehydration stresses. The relative expression levels of unstressed plants (0 h) were normalized to 1. One asterisk (*) indicates statistically significant differences between control (0 h) and stress-treated plants (*p* < 0.05); two asterisks (**) indicate statistically extremely significant differences between control (0 h) and stress-treated plants (*p* < 0.01). The black columns represent the salt treatment and the grey columns represent the dehydration treatment.

To screen sweet potato miRNAs participating in stress resistance and explore the potential functions of these twelve miRNAs under stress conditions, qRT-PCR was performed. Under the salt treatment, varying degrees of changes were observed in the expression of these twelve miRNAs (Figure 3). Weak induction was detected in miR156g, miR158a-3p and miR161.1 (Figure 3). The expression of miR157d, miR167d, miR397a and novel 120 was induced gradually and reached a maximum level at 12 h after the salt treatment; the expression level of miR167d increased by more than 20-fold (Figure 3). Novel 104 and novel 522 reached their maximum levels at 24 h after the salt treatment, and the expression level of novel 522 was increased by approximately 25-fold (Figure 3). In contrast, the expression of novel 140 and novel 359 was seriously inhibited by the salt treatment (Figure 3).

After the dehydration treatment, the great majority of miRNAs were upregulated significantly (Figure 3). The expression of miR156g, miR167d, novel 104, novel 120 and novel 214 was induced gradually from 1 h to 48 h of the PEG treatment (Figure 3). Among them, the levels of two miRNAs, miR167d and novel 120, were increased by more than 100-fold after 48 h the PEG treatment (Figure 3). miR157d and novel 359 were accumulated markedly after 1 h PEG treatment (Figure 3). Four miRNAs, miR158a-3p, miR161.1, miR397a and novel 140, exhibited their maximum induction after 24 h of the PEG treatment, and the expression of miR158a-3p was increased by more than 800-fold (Figure 3). The level of novel 522 was reduced by approximately 80% (Figure 3).

**Figure 4:**
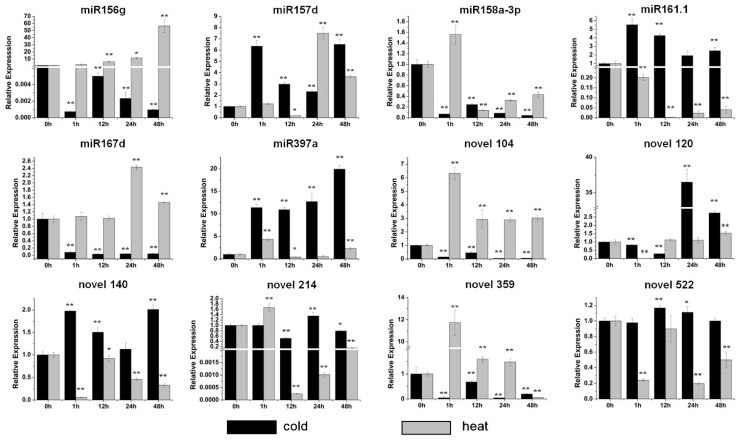
Expression profiles of six conserved and six novel miRNAs under cold and heat stresses. The relative expression levels of unstressed plants (0 h) were normalized to 1. One asterisk (*) indicates statistically significant differences between control (0 h) and stress-treated plants (*p* < 0.05), and two asterisks (**) indicate statistically extremely significant differences between control (0 h) and stress-treated plants (*p* < 0.01). The black columns represent the cold treatment and the grey columns represent the heat treatment.

The significant inhibition of the expression of five miRNAs, miR156g, miR158a-3p, miR167d, novel 104 and novel 359, was detected after the cold treatment (Figure 4). The expression of the other seven miRNAs was all increased, and the maximum induction was detected after 48 h of the cold treatment with miR397a (Figure 4). For the heat treatment the expression of most of the miRNAs was increased (Figure 4). miR156g was increased gradually from 1 h to 48 h, and reached a maximum value at 48 h with an approximately 60-fold increase under high temperature (Figure 4). miR158a-3p, miR397a, novel 104 and novel 359 were accumulated heavily after 1 h of heat treatment (Figure 4). The levels of miR157d and miR167d were increased dramatically after 24 h of heat treatment (Figure 4). The significant inhibition of miR161.1, novel 140, novel 214 and novel 522 was observed under heat treatment (Figure 4). Overall, the results indicated that these miRNAs are stress-responsive.

### 3.4. The Expression of Sweet Potato miRNAs Was Impacted by Multiple Hormones

**Figure 5:**
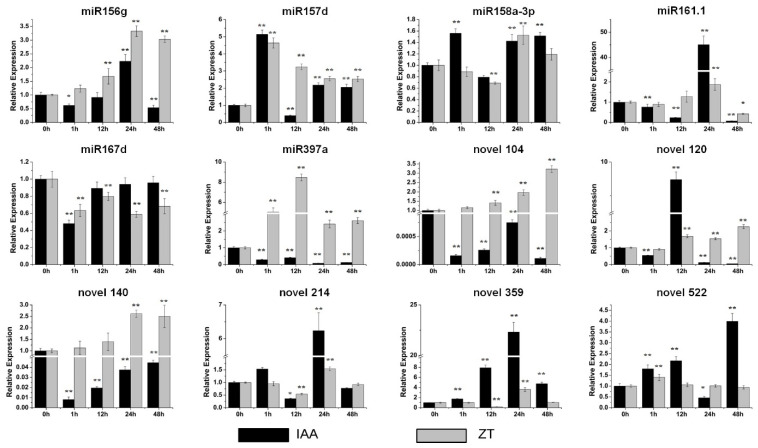
Expression profiles of six conserved and six novel miRNAs under the IAA (indole acetic acid) and ZT (zeaxanthin) treatments. The relative expression levels of unstressed plants (0 h) were normalized to 1. One asterisk (*) indicates statistically significant differences between control (0 h) and hormone-treated plants (*p* < 0.05), and two asterisks (**) indicate statistically extremely significant differences between control (0 h) and hormone-treated plants (*p* < 0.01). The black columns represent the IAA treatment and the grey columns represent the ZT treatment.

The expression of these miRNAs under stress-related and storage-root-development-related hormone treatments was investigated by qRT-PCR to further explore miRNA functions in stress responses and sweet potato development. For the IAA treatment, there were no obvious changes in miR158a-3p or miR167d (Figure 5), while miR156g, miR161.1, novel 214 and novel 359 showed their highest expression at 24 h of the IAA treatment (Figure 5). The most upregulated miRNA by IAA was miR161.1, which was upregulated by more than 45-fold (Figure 5). miR157d, novel 120 and novel 522 accumulated the highest at 1 h, 12 h and 48 h of the IAA treatment, respectively (Figure 5). In contrast, the levels of miR397a, novel 104 and novel 140 decreased dramatically (Figure 5). Five miRNAs, miR158a-3p, miR161.1, novel 120, novel 214 and novel 522, barely changed in response to the ZT treatment (Figure 5). The levels of miR156g and novel 359 increased the most at 24 h of the IAA treatment (Figure 5). Novel 104 and novel 140 were upregulated gradually and reached a maximum at 48 h of the IAA treatment (Figure 5). miR157d and miR397a showed their highest expression at 1 h and 12 h of the IAA treatment, respectively (Figure 5). The expression of miR167d was inhibited dramatically after the IAA treatment (Figure 5).

**Figure 6:**
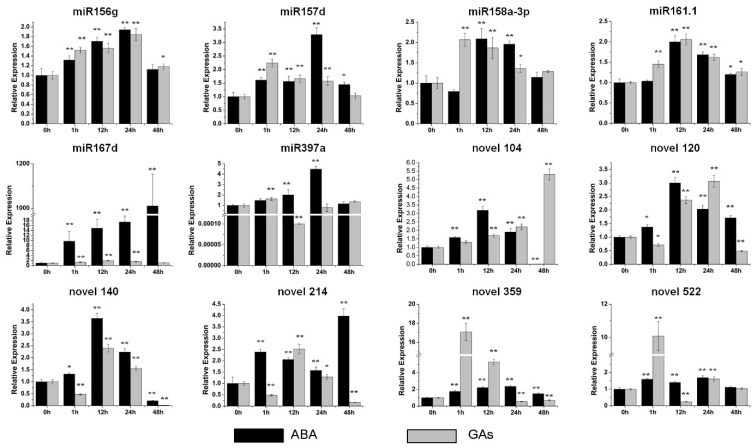
Expression profiles of six conserved and six novel miRNAs under the ABA (abscisic acid) and GAs (gibberellin) treatments. The relative expression levels of unstressed plants (0 h) were normalized to 1. One asterisk (*) indicates statistically significant differences between control (0 h) and hormone-treated plants (*p* < 0.05), and two asterisks (**) indicate statistically extremely significant differences between control (0 h) and hormone-treated plants (*p* < 0.01). The black columns represent the ABA treatment and the grey columns represent the GAs treatment.

Most of the miRNAs were upregulated by ABA and GAs. In particular, miR167d was the most induced miRNA under the ABA treatment and was up-regulated by more than 1000-fold compared with the control (Figure 6). Novel 359 was the most upregulated miRNA by GAs, with an increase of approximately 17-fold (Figure 6). Above all, these data indicated that the level of these miRNAs is impacted by multiple hormones.

### 3.5. The Target Prediction of the Twelve miRNAs

**Figure 7:**
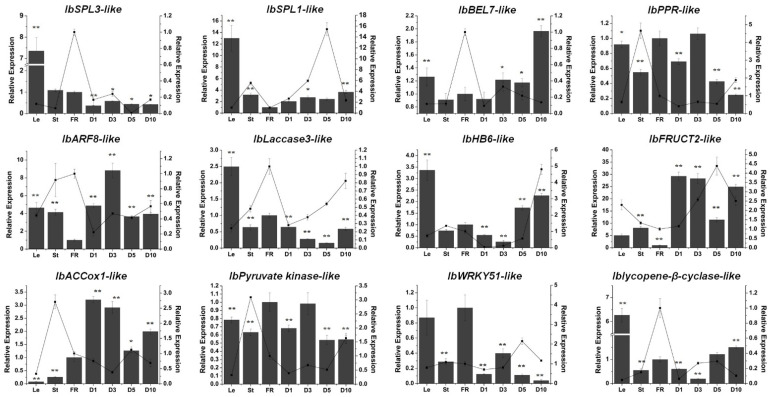
The potential targets of six conserved and six novel miRNAs. The relative expression levels of FR were normalized to 1. One asterisk (*) indicates statistically significant differences between FR and other tissues (*p* < 0.05), and two asterisks (**) indicate statistically extremely significant differences between FR and other tissues (*p* < 0.01). The tissues were obtained from Xushu 22. Le, leaves obtained from three-month-old plants; St, stems obtained from three-month-old plants; FR, fibrous roots; D1, 1 cm storage roots; D3, 3 cm storage roots; D5, 5 cm storage roots; and D10, 10 cm storage roots. The columns represent the potential target expression, and the lines represent the corresponding miRNA expression.

To predict the targets of these miRNAs, the RNA-seq and sRNA-seq data were combined and analyzed, and a set of potential targets of each miRNA were identified [20]. Then, qRT-PCR was performed to validate the predicted target expression. As with previous reports in other species, the potential targets of miR156g and miR157d were two SPL (squamosa promoter-binding-like) genes (*IbSPL3-like* and *IbSPL1-like*) (Figure 7). A BEL1-like gene (*IbBEL7-like*) was predicted to be a potential target of miR158a-3p (Figure 7). The expression of *IbARF8-like*, an ARF gene, showed a reverse trend compared with the miR167d expression profile (Figure 7). One PPR (pentatricopeptide repeat) gene (*IbPPR-like*) was predicted to be a potential target of miR161.1 (Figure 7). The candidate target of 397a was a laccase gene (*IbLaccase 3-like*) (Figure 7). Moreover, a homeobox-leucine zipper gene (*IbHB6-like*), a soluble acid invertase (*IbFRUCT2-like*), an ethylene biosynthesis gene 1-aminocyclopropane-1-carboxylate oxidase gene (*IbACCox1-like*), a pyruvate kinase gene (*IbPyruvate kinase-like*), a WRKY transcription factor gene (*IbWRKY51-like*) and a lycopene β cyclase gene (*Iblycopene-β-cyclase-like*) were predicted to be the candidate targets of novel 104, novel 120, novel 140, novel 214, novel 359 and novel 522, respectively (Figure 7).

## 4. Discussion

### 4.1. Twelve miRNAs May Play a Role in Storage Root Development of Sweet Potato

Much evidence of miRNAs being involved in development has been found. For instance, miR156, miR319 and miR775 have been confirmed to play a role in leaf development [6,23,24]. miR172 and miR156 play a role in flower initiation and the flowering of plants [7,25]. Several miRNAs, including miR160, miR390, miR393 and miR847, affect root formation and development [8,26,27,28]. A set of miRNAs, such as miR164 and miR165, have been proven to impact stem development [29,30]. However, there are few reports about the roles of miRNAs in storage root thickening. Here, we identified and characterized six conserved miRNAs and six new miRNAs from sweet potato (Table S1). Our expression analysis results showed that miR156g, miR158a-3p, miR167d and novel 522 were highly expressed in fibrous roots but exhibited low expression in storage roots (Figure 2). In contrast, the level of miR157d and novel 359 was much higher in storage roots than in fibrous roots (Figure 2). These results indicated that these miRNAs might participate in sweet potato storage root development.

The hormone treatments suggested that miR156g, miR157d and novel 359 were all upregulated by the primary thickening growth of storage-root-related hormones (IAA/ZT) [31,32] (Figure 5). In particular, the level of novel 359 was induced by more than 20-fold after the IAA treatment (Figure 5). In addition, the expression of miR158a-3p, miR167d and novel 522 was upregulated by the hormones that mainly play roles in the later stage of storage root development (ABA/GAs) [31,32] (Figure 6). Among them, miR167d was upregulated by approximately 1000-fold by ABA, and novel 522 was increased by approximately 10-fold by ZT (Figure 5).

Moreover, the potential targets of these miRNAs have been predicted by high-throughput sequencing; one predicted target of each miRNA has been chosen and its expression validated by qRT-PCR (Figure 7). Although the relationship between target genes and miRNAs requires further confirmation, these results provide some information for exploring the functions of these miRNAs. The results showed that two *SPLs*, the important lateral root formation regulators, were potential targets of miR156g and miR157d [33]. The target of miR158a-3p and novel 104 are two homeobox genes (Figure 7), which have been reported to be an important regulator in plant development, including root growth and development [34,35]. The potential targets of miR167d and novel 140 are related to hormone biosynthesis or response (Figure 7). The potential target of miR167d is an auxin response factor which has been shown to regulate root formation in other species [36]. *ACCox1*, a potential target of novel 140, is an ethylene biosynthesis gene, indicating that novel 140 is related to ethylene biosynthesis. Additionally, ethylene has been reported to be related to the formation and development of potato as well as sugar accumulation. Additionally, in order to further explore the function of these miRNAs, the sequences of these twelve mature miRNAs and pre-miRNAs have been screened in the genome of wild sweet potato species *I. trifida* and *I. triloba*, whose roots cannot thicken. The results showed that, apart from novel 522, the other eleven miRNAs can be identified, indicating that novel 522 may be a miRNA unique to sweet potato. Combining the tissue expression and hormone response results, we speculate that novel 522 may be an essential regulator in the formation and development of sweet potato storage roots. Altogether, these results suggest that these miRNAs might play crucial roles in storage root thickening and development.

### 4.2. miRNAs May Play a Role in Sweet Potato Abiotic Stress Responses

Plant miRNAs have a close relationship with various stress resistances. For example, miR164, miR394 and miR408 have been reported to be related to salt tolerance [9,10,37]. miR168, miR169 and miR319 are important regulators of the drought stress response [38,39,40]. Several miRNAs, including miR166 and miR319, play critical roles in cold/heat tolerance [41]. In addition, some miRNAs have been confirmed to help plants cope with oxidative and flood stresses [11,12].

Here, most miRNAs were shown to respond to abiotic stresses in sweet potato (Figure 3 and Figure 4). Among them, miR156g was distinctly induced by drought and high-temperature stresses (Figure 3 and Figure 4). miR167d was markedly induced by salt and drought stresses but significantly inhibited by cold stress (Figure 3 and Figure 4). miR158a-3p was dramatically upregulated by drought stress (Figure 3). Additionally, our results showed that novel 120 was significantly induced by drought and low-temperature stresses, and that the level of novel 214 increased sharply under salt stress treatment (Figure 3 and Figure 4). The three conserved miRNAs (miR156g, miR158a-3p and miR167d) have all been defined as stress responders in other species [33,42,43]. Furthermore, the candidate targets of these miRNAs have also been reported to be related to plant stress responses. *SPL3*, the target of miR156g, belongs to SBP box family, which is an important regulator in recurring environmental stress [33]. The candidate target of miR167d is an auxin response factor that is reported to be related to salinity tolerance and low-temperature resistance [36]. The expression of soluble acid invertase gene, the candidate targe of novel 120, has been reported to be impacted by salt, drought and heat stresses [44,45,46]. In addition, the overexpression of the lycopene β cyclase gene, a candidate target of novel 522, increased salt and drought tolerance [47]. Moreover, these five miRNAs were all induced by ABA, a crucial hormone modulator in regulating abiotic stresses (Figure 6). Above all, the data indicate that these miRNAs have functions in stress responses.

### 4.3. Some miRNAs May Play Roles in Abiotic Stress Responses and Storage Root Development of Sweet Potato Simultaneously

Sweet potato is a tuberous root crop with strong environmental stress resistance and tolerance of poor soil. Thus, it is beneficial to study the storage root formation and stress responses of sweet potato to identify the mechanisms involved. Plant miRNAs are essential regulators of both stress responses and plant development, and some miRNAs have been stated to play roles in both aspects. For instance, miR319 is critical for leaf and flower development, and simultaneously regulates salt and drought tolerances [6,13,48]. miR160 is an important regulator in root development, salt tolerance and heat tolerance [28,49]. Moreover, miR166 plays essential roles in shoot apical meristem development, leaf development and cold tolerance [30,41]. Our results showed that miR156g, miR158a-3p, miR157d, miR167d, novel 359 and novel 522 were differentially expressed in fibrous roots and storage roots, and simultaneously responded to one or more stresses (Figure 2, Figure 3 and Figure 4). In addition, these miRNAs were all impacted by storage-root-related or stress-response-related hormones (Figure 5 and Figure 6). Together, these results suggested that these miRNAs might simultaneously play roles in both the abiotic stress responses and storage root development of sweet potato.

Collectively, our results identified and thoroughly analyzed the stress- and storage-root-thickening-related miRNAs, which would provide useful information for the further study of miRNA functions and sweet potato breeding.

## Supplementary Materials

The following are available online at https://www.mdpi.com/article/10.3390/genes13010110/s1, Table S1: The sequences of the mature miRNAs and pre-miRNAs, Table S2: Primers used in qRT-PCR and reverse transcription of miRNAs.
